# Comparison of surgically induced astigmatism between anterior and total cornea in 2.2 mm steep meridian incision cataract surgery

**DOI:** 10.1186/s12886-021-02131-x

**Published:** 2021-10-19

**Authors:** Young-chae Yoon, Minji Ha, Woong-Joo Whang

**Affiliations:** Department of Ophthalmology, Yeouido St. Mary’s Hospital, College of Medicine, The Catholic University of Korea, #10 63ro, Youngdeungpo-Gu, Seoul, 06576 Republic of Korea

## Abstract

**Background:**

This study aimed to compare surgically induced astigmatism (SIA) on the anterior and total cornea during cataract surgery through a 2.2 mm steep meridian incision.

**Methods:**

The study included 69 left eyes of 69 patients who had undergone cataract surgery. The 69 eyes were classified into three subgroups according to the preoperative steep meridian. Following phacoemulsification, an intraocular lens was inserted into the bag. The keratometric measurements were taken 12 months postoperatively, on the anterior cornea (automated keratometer and anterior keratometry [K] from a rotating Scheimpflug camera) and total cornea (equivalent K reading [EKR] 3.0 mm, EKR 4.5 mm, total corneal refractive power (TCRP) 2.0 mm ring, TCRP 3.0 mm zone, TCRP 4.0 mm zone). The SIA was analyzed for each parameter.

**Results:**

On the double-angle polar plot, the summated vector mean values of SIA determined by the automated keratometer and Scheimpflug anterior K were 0.28 diopter (axis: 177°) and 0.37 diopter (axis: 175°) in with-the-rule (WTR) astigmatism; 0.03 diopter (axis: 156°) and 0.18 diopter (axis: 177°) in oblique astigmatism; 0.15 diopter (axis: 96°) and 0.17 diopter (axis: 73°) in against-the-rule (ATR) astigmatism. The mean SIAs on the total cornea ranged from 0.31 to 0.42 diopter in WTR astigmatism; from 0.16 to 0.27 diopter in oblique astigmatism; from 0.04 to 0.11 diopter in ATR astigmatism. Mean magnitude SIA ranged from 0.41 to 0.46 diopter on anterior corneal surface and 0.50 to 0.62 diopter on total cornea. J_0_ and J_45_ of the posterior cornea showed no significant changes after cataract surgery, and the changes in J_0_ and J_45_ did not show any statistical differences between the anterior and total cornea (all *p* > 0.05).

**Conclusions:**

There were no differences in the summed vector mean values of SIA between the anterior cornea and the total cornea.

## Background

In modern cataract surgery, complications are uncommon, and the importance of refractive outcomes is increasing [1]. Consequently, minimizing residual astigmatism is one of the main goals of modern cataract surgery. It is important to calculate surgically induced astigmatism (SIA) accurately in the process of astigmatic correction.

The proper choice of incision location in cataract surgery helps reduce astigmatism. The clear corneal incision located on the preoperative steep meridian decreased keratometric astigmatism at the sup, superotemporal, and temporal locations [2–4]. The accurate assessment of SIA is also necessary for surgical procedures, such as toric IOL implantation and astigmatic keratotomy combined with cataract surgery. SIA is included as part of the toric IOL nomogram [5] or the arcuate keratotomy nomogram. Jin et al. [6] found that 0.5 diopter of SIA would be added by a 2.8-mm wide superior incision, whereas Goggin et al. [7, 8] found that 0.62 diopter of SIA would be added by a 2.2–2.3 mm wide superior incision. Alio et al. [9] applied 0.5 diopter of SIA after a 2.7-mm-wide steep meridian incision. Zhang et al. [10] recently compared residual astigmatism according to keratometric measurements and concluded that the 0.2 diopter of SIA is induced by a temporal incision.

Ofir et al. [11] found that there were differences in the calculated SIA depending on the type of keratometer used. SIA on the posterior corneal surface was also found to be an important factor that had a clinical impact on the assessment of astigmatism [12]. However, most previous studies investigating SIA only considered the anterior corneal surface [2, 5, 13–18]. It is also important for cataract surgeons to know whether the posterior cornea has an effect on enhancing or reducing the SIA of the anterior cornea. Additionally, no previous study has calculated SIA on the total cornea after 2.2-mm micro coaxial cataract surgery on the steep axis.

In this study, we aimed to evaluate the SIA using an automated keratometer and a Scheimpflug rotating camera. SIAs on the total cornea were also analyzed using a Scheimpflug camera. The SIAs calculated using various keratometric measurements were compared.

## Methods

### Participants

This retrospective study included 69 left eyes of 69 patients who underwent cataract surgery at our institution between September 2017 and August 2018. This study was approved by the Institutional Review Board Committee of Yeouido St. Mary Hospital, and informed consent was obtained from all patients before commencement. The study adhered to the tenets of the Declaration of Helsinki for the use of human participants in biomedical research. None of the patients had a history of ocular disease, previous ocular surgery, or general disorders that affected the cornea, and there were no intraoperative complications.

### Cataract surgery

After preoperative measurements, all patients underwent cataract surgery through a 2.2 mm micro coaxial incision. All surgeries were performed using the OZil torsional handpiece with the Centurion System (Alcon, Fort Worth, TX, USA). All procedures were performed by a single surgeon (W. J. W.). Local anesthesia was administered using 0.5% proparacaine hydrochloride (Alcaine, Alcon). Surgery was performed through a self-sealing, clear corneal incision on the steep meridian provided by Scheimpflug anterior keratometry (K) to reduce keratometric astigmatism [3, 19, 20]. With the patient seated, the corneal limbus was marked at the 0°, 180°, and 270° axes using pre-op toric reference marker (ASICO, IL, USA). Next, with the patient lying on the surgical table, the steep meridian was identified and marked using a Mendez degree gauge (Katena Eye Inc., NJ, USA) with the aid of preplaced reference points. The Intrepid ClearCut 2.2 mm dual-bevel metal keratome (Alcon, Fort Worth, Texas) was used to make a two-step 2.2 mm incision. Using a Beaver blade, a 1.0 mm single-plane side-port clear corneal incision was made 90° to the left of the main corneal incision. The cataract was removed using a 0.9 mm mini flared 30-degree Kelman ABS tip with a 2.2 mm micro coaxial incision. Phacoemulsification was performed with 100% torsional ultrasound, a vacuum of 350 mmHg, and an aspiration rate of 35 cc/min. Following phacoemulsification, an intraocular lens (IOL; Johnson & Johnson ZCB00, Santa Ana, USA) was inserted into the bag. At the end of the surgery, the incision sites were hydrated with a balanced salt solution and no sutures were applied.

### Keratometric measurements

The keratometric values were measured preoperatively and 12 months postoperatively using an RK-5 automated keratometer (Canon, Tochigiken, Japan) and Pentacam rotating Scheimpflug camera (Oculus, Wetzler, Germany). The Pentacam HR analyzed the cornea via 25 picture scans, and only scans that had an examination quality specification graded by the instrument as “OK” were included in this study.

The average K reading was the arithmetic mean of the pair of meridians, 90° apart, showing the greatest difference in axial power within the central 3.0 mm. This was equivalent to the simulated K of traditional corneal topography and was calculated by entering the corneal curvature radius into the thin-lens formula for paraxial imagery, which considers the cornea as a single refractive sphere.

The equivalent K reading (EKR) was calculated using the formula previously described by Holladay et al. [21], taking into account the effect of the posterior corneal curvature.

The total corneal refractive power (TCRP) was calculated using the ray tracing method. Corneal thickness and curvatures of both the anterior and posterior corneal surfaces were obtained using Scheimpflug imaging. Snell’s law and the specific refractive indices of air, cornea, and aqueous humor were used to calculate the corneal power.

### Data analysis

A total of 69 eyes were classified into three subgroups based on the steep meridian measured by Scheimpflug anterior K. Thirty-two eyes were defined as with-the-rule (WTR) astigmatism with a steep meridian between 60° and 120°. Twenty-four eyes were classified as against-the-rule (ATR) astigmatism with a steep meridian between 150° and 30°. The remaining 13 eyes were classified into the oblique astigmatism group.

Two parameters were used to analyze the SIA: (1) the mean magnitude SIA [22] and (2) the summed mean values of SIA [2, 23, 24]. SIA was determined as the vectorial difference between the preoperative and postoperative astigmatism, and the mean magnitude SIA was defined as the mean absolute value of each vector. The preoperative and postoperative corneal astigmatism with their constituent magnitude and meridian were decomposed to J_0_ (vertical and horizontal components of corneal astigmatism transposed in the Jackson coefficient orthogonal system) and J_45_ (an oblique component of corneal astigmatism transposed in the Jackson coefficient orthogonal system). ∆ J_0_ was defined as the value obtained by subtracting the preoperative J_0_ from the postoperative J_0_, and ∆ J_45_ was defined as the value obtained by subtracting the preoperative J_45_ from the postoperative J_45._ The mean centroid SIA was calculated using Eye Pro 2013: Astig PLOT (for iPhone/iPad [Apple, Cupertino, CA, USA]), developed by Dr. Edmondo Borasio and was represented by double-angle polar plots [2]. The flattening effect providing the astigmatic change at the steep meridian and torque rotating preoperative astigmatism were also calculated [5, 25].

### Statistical analysis

Statistical analysis was performed using SPSS statistical software (version 23.0, SPSS, Inc., Chicago, IL, USA). The normality of the distribution for each data point was checked using the Kolmogorov-Smirnov test. The paired t-test and the Wilcoxon ranked-sum test were used to compare preoperative corneal astigmatism with postoperative corneal astigmatism and to determine if there were significant differences between the SIA on the anterior cornea and the SIA on the total cornea. Statistical significance was set at *p* <  0.05.

## Results

A total of 69 left eyes (69 patients) were evaluated in this study. The mean age was 63.39 ± 8.17 years (range: 46–83 years). There were 43 female patients (62.3%).

Table 1 presents the preoperative and postoperative data. The mean corneal astigmatism measured by automated keratometer, Scheimpflug anterior K, EKR 4.5 mm zone, and TCRP 4.0 mm zone showed significant changes at 3 months postoperatively (all *p* <  0.01). Table 2 shows the preoperative and postoperative data for the three subgroups. In the WTR astigmatism group, the mean corneal astigmatism by automated keratometer, Scheimpflug anterior K, EKR 4.5 mm zone, and TCRP 4.0 mm zone were significantly reduced (all *p* <  0.01). In contrast, in the oblique astigmatism and ATR astigmatism groups, only the mean corneal astigmatism measured with Scheimpflug anterior K was significantly decreased (*p* = 0.037, for the oblique astigmatism group and *p* = 0.021, for the ATR astigmatism group).

**Table 1:** Mean corneal power, mean magnitude of astigmatism and mean value of J_0_ & J_45_ in total 69 eyes

Keratometric measurement	Preoperative (Mean diopter ± SD)				Postoperative (Mean diopter ± SD)				* *p* value			
	corneal power	astigmatism			corneal power	astigmatism			corneal power	astigmatism		
		magnitude	J_0_	J_45_		magnitude	J_0_	J_45_		magnitude	J_0_	J_45_
Automated K	43.68 ± 1.51	0.81 ± 0.75	−0.11 ± 0.50	0.04 ± 0.21	43.68 ± 1.50	0.71 ± 0.60	−0.07 ± 0.42	0.03 ± 0.20	0.84	0.008	0.06	0.66
Scheimpflug Anterior K	43.66 ± 1.50	0.89 ± 0.75	−0.15 ± 0.52	0.03 ± 0.22	43.64 ± 1.67	0.70 ± 0.60	−0.07 ± 0.41	0.03 ± 0.19	0.65	< 0.001	0.003	0.96
Scheimpflug Posterior K	−6.39 ± 0.28	0.30 ± 0.73	0.14 ± 0.09	−0.02 ± 0.05	− 6.40 ± 0.23	0.31 ± 0.18	0.14 ± 0.09	−0.03 ± 0.06	0.43	0.27	0.53	0.30
Scheimpflug EKR 3.0-mm	43.47 ± 1.49	0.81 ± 0.70	−0.01 ± 0.49	0.00 ± 0.24	43.45 ± 1.46	0.73 ± 0.54	0.07 ± 0.40	−0.01 ± 0.21	0.87	0.14	0.025	0.61
Scheimpflug EKR 4.5-mm	43.68 ± 1.46	0.81 ± 0.60	−0.03 ± 0.46	0.01 ± 0.22	43.71 ± 1.49	0.69 ± 0.55	0.03 ± 0.39	0.00 ± 0.20	0.28	0.005	0.023	0.61
Scheimpflug TCRP 2.0-mm ring	42.90 ± 1.63	0.94 ± 0.79	−0.02 ± 0.55	0.00 ± 0.29	42.90 ± 1.64	0.83 ± 0.61	0.10 ± 0.46	0.00 ± 0.22	0.35	0.10	0.003	0.78
Scheimpflug TCRP 3.0-mm zone	42.93 ± 1.62	0.90 ± 0.71	−0.01 ± 0.52	0.01 ± 0.26	42.93 ± 1.63	0.80 ± 0.59	0.09 ± 0.44	0.00 ± 0.21	0.45	0.10	0.002	0.82
Scheimpflug TCRP 4.0-mm zone	43.03 ± 1.60	0.86 ± 0.64	−0.03 ± 0.49	0.00 ± 0.23	43.04 ± 1.63	0.71 ± 0.59	0.06 ± 0.42	0.00 ± 0.19	0.31	< 0.001	0.002	0.85

*SD* standard deviation

* *p*-value by paired t-test

**Table 2:** Mean corneal power, mean magnitude of astigmatism and mean value of J_0_ & J_45_ in 3 subgroups classified according to preoperative steep axis (WTR: 32 eyes; oblique: 13 eyes; ATR: 24 eyes)

Keratometric measurement		Preoperative (Mean diopter ± SD)				Postoperative (Mean diopter ± SD)				* p value			
		corneal power	astigmatism			corneal power	astigmatism			corneal power	astigmatism		
			magnitude	J_0_	J_45_		magnitude	J_0_	J_45_		magnitude	J_0_	J_45_
WTR	Automated K	43.65 ± 1.59	1.01 ± 0.92	−0.46 ± 0.46	0.05 ± 0.21	43.65 ± 1.58	0.85 ± 0.74	−0.32 ± 0.40	0.03 ± 0.22	0.76	0.009	< 0.001	0.75
	Scheimpflug Anterior K	43.64 ± 1.59	1.10 ± 0.91	−0.48 ± 0.48	0.07 ± 0.22	43.60 ± 1.54	0.81 ± 0.72	−0.30 ± 0.40	0.04 ± 0.22	0.76	< 0.001	< 0.001	0.39
	Scheimpflug Posterior K	−6.39 ± 0.20	0.38 ± 0.17	0.18 ± 0.09	−0.03 ± 0.05	− 6.39 ± 0.18	0.38 ± 0.16	0.18 ± 0.08	−0.03 ± 0.06	0.62	0.97	0.72	0.78
	Scheimpflug EKR 3.0-mm	43.48 ± 1.58	0.85 ± 0.90	−0.29 ± 0.50	− 0.01 ± 0.23	43.45 ± 1.50	0.71 ± 0.63	−0.12 ± 0.40	−0.03 ± 0.23	0.91	0.28	0.002	0.61
	Scheimpflug EKR 4.5-mm	43.61 ± 1.51	0.90 ± 0.74	−0.33 ± 0.43	0.00 ± 0.22	43.63 ± 1.57	0.68 ± 0.63	−0.17 ± 0.37	0.00 ± 0.22	0.59	0.004	< 0.001	0.94
	Scheimpflug TCRP 2.0-mm ring	42.94 ± 1.80	0.97 ± 1.04	−0.36 ± 0.55	0.02 ± 0.27	42.86 ± 1.69	0.75 ± 0.67	−0.15 ± 0.42	0.00 ± 0.24	0.79	0.13	< 0.001	0.90
	Scheimpflug TCRP 3.0-mm zone	42.96 ± 1.79	0.91 ± 0.92	−0.33 ± 0.50	0.02 ± 0.25	42.88 ± 1.69	0.72 ± 0.67	−0.15 ± 0.41	0.00 ± 0.23	0.85	0.12	< 0.001	0.72
	Scheimpflug TCRP 4.0-mm zone	43.03 ± 1.77	0.92 ± 0.82	−0.34 ± 0.46	0.02 ± 0.23	42.97 ± 1.72	0.66 ± 0.68	−0.17 ± 0.39	0.01 ± 0.21	0.97	< 0.001	< 0.001	0.60
oblique	Automated K	43.81 ± 1.32	0.54 ± 0.27	−0.04 ± 0.08	0.01 ± 0.30	43.79 ± 1.35	0.52 ± 0.24	−0.03 ± 0.16	0.00 ± 0.24	0.74	0.66	0.86	0.70
	Scheimpflug Anterior K	43.81 ± 1.29	0.70 ± 0.27	−0.11 ± 0.18	0.06 ± 0.32	43.83 ±1.32	0.59 ± 0.22	−0.02 ± 0.21	0.05 ± 0.24	0.97	0.037	0.06	0.60
	Scheimpflug Posterior K	−6.46 ± 0.28	0.29 ± 0.15	0.13 ± 0.07	−0.01 ± 0.08	−6.49 ± 0.33	0.31 ± 0.17	0.12 ± 0.10	−0.04 ± 0.07	0.44	0.53	0.86	0.06
	Scheimpflug EKR 3.0-mm	43.47 ± 1.33	0.65 ± 0.29	0.02 ± 0.16	0.07 ± 0.32	43.49 ± 1.16	0.65 ± 0.37	0.09 ± 0.26	0.04 ± 0.26	0.94	0.81	0.35	0.55
	Scheimpflug EKR 4.5-mm	43.78 ± 1.32	0.65 ± 0.26	0.00 ± 0.20	0.08 ± 0.29	43.82 ± 1.24	0.58 ± 0.28	0.07 ± 0.20	0.02 ± 0.26	0.99	0.38	0.28	0.42
	Scheimpflug TCRP 2.0-mm ring	42.83 ± 1.47	0.82 ± 0.30	0.05 ± 0.19	0.08 ± 0.40	42.96 ± 1.61	0.82 ± 0.34	0.16 ± 0.32	0.01 ± 0.28	0.18	0.91	0.15	0.20
	Scheimpflug TCRP 3.0-mm zone	42.86 ± 1.45	0.79 ± 0.28	0.02 ± 0.19	0.09 ± 0.38	42.99 ± 1.61	0.78 ± 0.29	0.14 ± 0.29	0.04 ± 0.28	0.24	0.96	0.10	0.38
	Scheimpflug TCRP 4.0-mm zone	43.05 ± 1.37	0.72 ± 0.19	0.01 ± 0.19	0.06 ± 0.33	43.14 ± 1.52	0.62 ± 0.21	0.11 ± 0.22	0.02 ± 0.23	0.20	0.17	0.06	0.38
ATR	Automated K	43.64 ± 1.54	0.70 ± 0.63	0.32 ± 0.31	0.05 ± 0.15	43.65 ± 1.54	0.64 ± 0.52	0.24 ± 0.31	0.04 ± 0.13	0.97	0.35	0.10	0.88
	Scheimpflug Anterior K	43.59 ± 1.53	0.71 ± 0.62	0.28 ± 0.35	−0.03 ± 0.15	43.59 ± 1.59	0.61 ± 0.55	0.21 ± 0.33	0.02 ± 0.13	0.78	0.021	0.024	0.17
	Scheimpflug Posterior K	−6.35 ± 0.25	0.19 ± 0.14	0.08 ± 0.08	−0.02 ± 0.04	−6.35 ± 0.23	0.22 ± 0.18	0.10 ± 0.09	−0.02 ± 0.05	0.87	0.19	0.11	0.93
	Scheimpflug EKR 3.0-mm	43.45 ± 1.50	0.85 ± 0.54	0.34 ± 0.32	−0.03 ± 0.18	43.44 ± 1.59	0.79 ± 0.51	0.32 ± 0.31	−0.02 ± 0.16	0.81	0.35	0.63	0.99
	Scheimpflug EKR 4.5-mm	43.71 ± 1.51	0.79 ± 0.51	0.34 ± 0.29	−0.02 ± 0.15	43.77 ± 1.56	0.76 ± 0.56	0.29 ± 0.35	−0.02 ± 0.14	0.31	0.50	0.14	0.95
	Scheimpflug TCRP 2.0-mm ring	42.88 ± 1.51	0.97 ± 0.60	0.39 ± 0.36	−0.05 ± 0.22	42.92 ± 1.65	0.94 ± 0.63	0.39 ± 0.38	−0.02 ± 0.17	0.66	0.33	0.75	0.49
	Scheimpflug TCRP 3.0-mm zone	42.92 ± 1.53	0.95 ± 0.57	0.39 ± 0.35	−0.06 ± 0.18	42.95 ± 1.66	0.91 ± 0.61	0.39 ± 0.36	−0.02 ± 0.15	0.72	0.44	0.67	0.57
	Scheimpflug TCRP 4.0-mm zone	43.00 ± 1.54	0.86 ± 0.52	0.38 ± 0.31	−0.05 ± 0.14	43.08 ± 1.61	0.82 ± 0.60	0.35 ± 0.34	−0.01 ± 0.13	0.32	0.44	0.39	0.36

*SD* standard deviation

* *p*-value by paired t-test

Table 3 shows the arithmetic mean SIA, change in J_0_ (∆ J_0_), and change in J_45_ (∆ J_45_) as determined by each type of keratometric measurement. The arithmetic mean SIAs determined by the automated keratometer and Pentacam anterior K were 0.41 and 0.46 diopter, respectively, whereas the SIA on the posterior cornea was 0.13 diopter. The SIA determined using the TCRP 2.0 mm ring was 0.68 diopter, which was the largest among the SIAs on the total cornea. This was followed by the SIA determined by the EKR 3.0 mm zone, TCRP 3.0 mm zone, EKR 4.5 mm zone, and TCRP 4.0 mm zone. In our comparison of arithmetic mean SIAs on the anterior cornea, the values determined by the automated keratometer and Scheimpflug anterior K were not significantly different (*p* = 0.09). The arithmetic mean SIAs of the anterior cornea were significantly higher than the mean arithmetic SIAs on the posterior cornea (all *p* <  0.001) but were less than the arithmetic mean SIAs on the total cornea except for the TCRP 4.0 mm zone (*p* = 0–0.021). However, the ∆ J_0_ and ∆ J_45_ values measured on the anterior corneal surface and the total cornea were not different (all *p* > 0.05). The arithmetic mean SIA, ∆ J_0_, and ∆ J_45_ classified according to the preoperative steep corneal meridian are listed in Table 4. The arithmetic mean SIA analyzed in the TCRP 4.0 mm zone showed no significant difference from the mean arithmetic SIAs calculated on the anterior corneal surface. In the case of ∆ J_0_ and ∆ J_45_, there was no statistically significant difference between the changes measured on the anterior surface of the cornea and those of the whole cornea, irrespective of the preoperative steep corneal meridian and the location of the corneal incision.

**Table 3:** Surgically induced astigmatism (SIA) determined using different keratometric measurement approaches. Arithmetic SIAs were calculated by the vector method

	Arithmetic SIA					∆J_0_			∆J_45_			Flattening (diopter)	Torque (dioter)
	Mean diopter ± SD	Range (diopter)	Median (diopter)	* p value	** *p* value	Mean diopter ± SD	* p value	** p value	Mean diopter ± SD	* p value	** p value		
Automated K	0.41 ± 0.27	0–1.44	0.35		0.09	0.04 ± 0.20		0.08	−0.02 ± 0.14		0.50	0.23 ± 0.31	−0.01 ± 0.29
Scheimpflug Anterior K	0.46 ± 0.30	0–1.63	0.43	0.09		0.08 ± 0.20	0.08		0.00 ± 0.17	0.50		0.34 ± 0.32	0.05 ± 0.30
Scheimpflug Posterior K	0.13 ± 0.07	0–0.29	0.11	< 0.001	< 0.001	0.01 ± 0.06	0.17	0.02	−0.01 ± 0.05	0.94	0.64	0.01 ± 0.11	−0.01 ± 0.10
Scheimpflug EKR 3.0-mm	0.62 ± 0.35	0.10–2.18	0.60	< 0.001	< 0.001	0.08 ± 0.27	0.33	0.99	−0.01 ± 0.23	0.92	0.49	0.34 ± 0.49	0.03 ± 0.39
Scheimpflug EKR 4.5-mm	0.52 ± 0.28	0.02–1.16	0.51	0.010	0.021	0.07 ± 0.22	0.42	0.28	−0.01 ± 0.19	0.79	0.52	0.32 ± 0.38	−0.02 ± 0.33
Scheimpflug TCRP 2.0-mm ring	0.68 ± 0.46	0–2.64	0.61	< 0.001	< 0.001	0.12 ± 0.32	0.06	0.11	−0.01 ± 0.24	0.96	0.46	0.40 ± 0.53	0.07 ± 0.48
Scheimpflug TCRP 3.0-mm zone	0.61 ± 0.37	0.06–2.08	0.55	< 0.001	< 0.001	0.11 ± 0.27	0.07	0.17	−0.01 ± 0.21	0.98	0.38	0.32 ± 0.50	0.06 ± 0.39
Scheimpflug TCRP 4.0-mm zone	0.50 ± 0.30	0.01–1.39	0.46	0.017	0.07	0.09 ± 0.22	0.15	0.96	0.00 ± 0.17	0.61	0.79	0.32 ± 0.40	0.03 ± 0.28

*SD* standard deviation

* *p*-value for comparison with Automated K

** *p*-value for comparison with Scheimpflug Anterior K

**Table 4:** Surgically induced astigmatism (SIA) determined using different keratometric measurement approaches in 3 subgroups classified according to preoperative steep axis. (WTR: 32 eyes; oblique: 13 eyes; ATR: 24 eyes) Arithmetic SIAs were calculated by the vector method

		Arithmetic SIA					∆J_0_			∆J_45_			Flattening (diopter)	Torque (dioter)
		Mean diopter ± SD	Range (diopter)	Median (diopter)	* p value	** p value	Mean diopter ± SD	* p value	** p value	Mean diopter ± SD	* p value	** p value		
WTR	Automated K	0.43 ± 0.33	0–1.44	0.36		0.07	0.14 ± 0.16		0.06	−0.02 ± 0.17		0.65	0.29 ± 0.34	0.00 ± 0.31
	Scheimpflug Anterior K	0.54 ± 0.37	0–1.63	0.47	0.07		0.18 ± 0.20	0.06		−0.03 ± 0.19	0.65		0.42 ± 0.39	0.02 ± 0.33
	Scheimpflug Posterior K	0.12 ± 0.07	0–0.27	0.11	< 0.001	< 0.001	0.00 ± 0.06	< 0.001	< 0.001	0.00 ± 0.04	0.79	0.50	0.02 ± 0.10	0.01 ± 0.10
	Scheimpflug EKR 3.0-mm	0.69 ± 0.44	0.11–2.18	0.74	0.001	0.002	0.17 ± 0.27	0.53	0.50	−0.02 ± 0.25	0.97	0.70	0.47 ± 0.54	0.02 ± 0.39
	Scheimpflug EKR 4.5-mm	0.58 ± 0.30	0.08–1.13	0.59	0.026	0.09	0.16 ± 0.20	0.58	0.14	0.00 ± 0.21	0.78	0.33	0.42 ± 0.39	0.00 ± 0.30
	Scheimpflug TCRP 2.0-mm ring	0.71 ± 0.51	0.14–2.64	0.62	< 0.001	< 0.001	0.21 ± 0.31	0.09	0.38	−0.01 ± 0.24	0.99	0.79	0.52 ± 0.61	−0.02 ± 0.35
	Scheimpflug TCRP 3.0-mm zone	0.67 ± 0.42	0.06–2.08	0.65	< 0.001	0.002	0.18 ± 0.27	0.22	0.99	−0.02 ± 0.23	0.90	0.96	0.47 ± 0.53	−0.01 ± 0.35
	Scheimpflug TCRP 4.0-mm zone	0.54 ± 0.34	0.01–1.39	0.58	0.06	0.67	0.17 ± 0.20	0.35	0.39	−0.01 ± 0.19	0.84	0.72	0.43 ± 0.42	0.00 ± 0.23
oblique	Automated K	0.37 ± 0.19	0.07–0.69	0.42		0.38	0.01 ± 0.19		0.13	−0.01 ± 0.10		0.92	0.17 ± 0.17	−0.10 ± 0.34
	Scheimpflug Anterior K	0.43 ± 0.20	0.10–0.67	0.50	0.38		0.09 ± 0.15	0.13		−0.01 ± 0.17	0.92		0.27 ± 0.18	0.11 ± 0.34
	Scheimpflug Posterior K	0.13 ± 0.09	0.01–0.26	0.11	< 0.001	< 0.001	0.00 ± 0.05	0.86	0.08	−0.03 ± 0.05	0.92	0.97	0.01 ± 0.09	−0.07 ± 0.11
	Scheimpflug EKR 3.0-mm	0.60 ± 0.25	0.20–0.96	0.60	0.055	0.035	0.07 ± 0.26	0.75	0.75	−0.04 ± 0.19	0.70	0.42	0.21 ± 0.41	0.06 ± 0.48
	Scheimpflug EKR 4.5-mm	0.49 ± 0.24	0.06–0.84	0.51	0.13	0.046	0.07 ± 0.26	0.46	0.86	−0.06 ± 0.16	0.13	0.20	0.25 ± 0.31	−0.03 ± 0.40
	Scheimpflug TCRP 2.0-mm ring	0.70 ± 0.29	0.20–1.31	0.69	0.005	0.001	0.11 ± 0.30	0.22	0.70	−0.07 ± 0.22	0.17	0.42	0.28 ± 0.43	0.23 ± 0.54
	Scheimpflug TCRP 3.0-mm zone	0.60 ± 0.21	0.19–0.94	0.65	0.019	0.002	0.12 ± 0.21	0.15	0.51	−0.05 ± 0.21	0.42	0.22	0.22 ± 0.29	0.23 ± 0.48
	Scheimpflug TCRP 4.0-mm zone	0.48 ± 0.19	0.24–0.79	0.52	0.09	0.09	0.10 ± 0.17	0.12	0.86	−0.04 ± 0.18	0.25	0.38	0.27 ± 0.24	0.12 ± 0.38
ATR	Automated K	0.39 ± 0.21	0.05–0.99	0.35		0.93	−0.07 ± 0.18		0.98	−0.02 ± 0.12		0.06	0.19 ± 0.33	0.01 ± 0.24
	Scheimpflug Anterior K	0.38 ± 0.21	0.10–0.88	0.33	0.93		−0.07 ± 0.14	0.98		0.05 ± 0.15	0.06		0.27 ± 0.24	0.05 ± 0.25
	Scheimpflug Posterior K	0.13 ± 0.07	0–0.29	0.11	< 0.001	< 0.001	0.02 ± 0.06	0.034	0.009	0.00 ± 0.05	0.93	0.12	−0.00 ± 0.12	−0.01 ± 0.09
	Scheimpflug EKR 3.0-mm	0.55 ± 0.26	0.15–1.17	0.47	0.043	0.006	−0.02 ± 0.22	0.55	0.42	0.01 ± 0.21	0.80	0.38	0.23 ± 0.44	0.01 ± 0.35
	Scheimpflug EKR 4.5-mm	0.45 ± 0.27	0.02–1.16	0.41	0.48	0.25	−0.05 ± 0.19	0.80	0.93	0.00 ± 0.18	0.84	0.20	0.22 ± 0.37	−0.04 ± 0.32
	Scheimpflug TCRP 2.0-mm ring	0.63 ± 0.49	0.14–2.36	0.47	0.08	0.009	0.01 ± 0.32	0.61	0.15	0.04 ± 0.24	0.35	0.80	0.30 ± 0.44	0.11 ± 0.59
	Scheimpflug TCRP 3.0-mm zone	0.53 ± 0.37	0.10–1.74	0.38	0.21	0.032	0.00 ± 0.26	0.61	0.06	0.03 ± 0.19	0.55	0.51	0.18 ± 0.50	0.05 ± 0.38
	Scheimpflug TCRP 4.0-mm zone	0.44 ± 0.28	0.13–1.19	0.40	0.53	0.17	−0.07 ± 0.21	0.46	0.38	0.04 ± 0.15	0.25	0.61	0.19 ± 0.41	0.02 ± 0.29

*SD* standard deviation

* *p*-value for comparison with Automated K in each group

** *p*-value for comparison with Scheimpflug Anterior K in each group

**Fig. 1:**
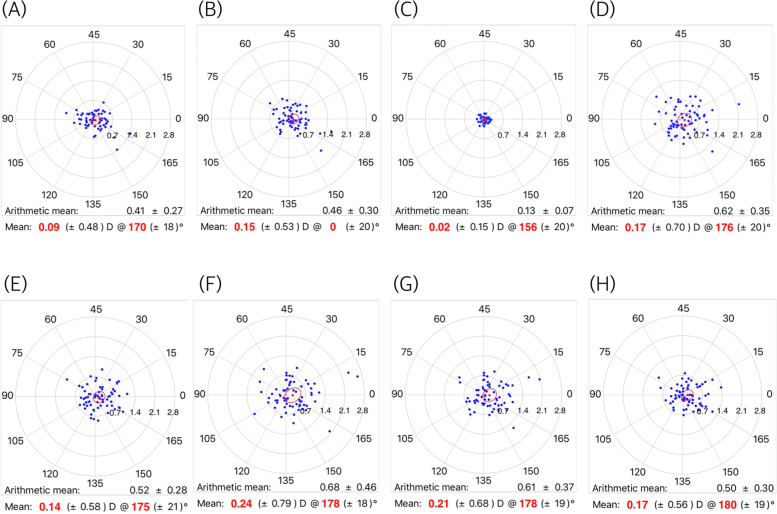
Surgically induced astigmatism (SIA) was calculated using each keratometric measurement. Each vector and the mean SIA are represented on a double-angle polar plot. **A** automated keratometer, **B** Scheimpflug anterior K, **C** Scheimpflug posterior K, **D** Scheimpflug EKR 3.0 mm, **E** Scheimpflug EKR 4.5 mm, **F** Scheimpflug TCRP 2.0 mm ring, **G** Scheimpflug TCRP 3.0 mm zone, and **H** Scheimpflug TCRP 4.0 mm zone

**Fig. 2:**
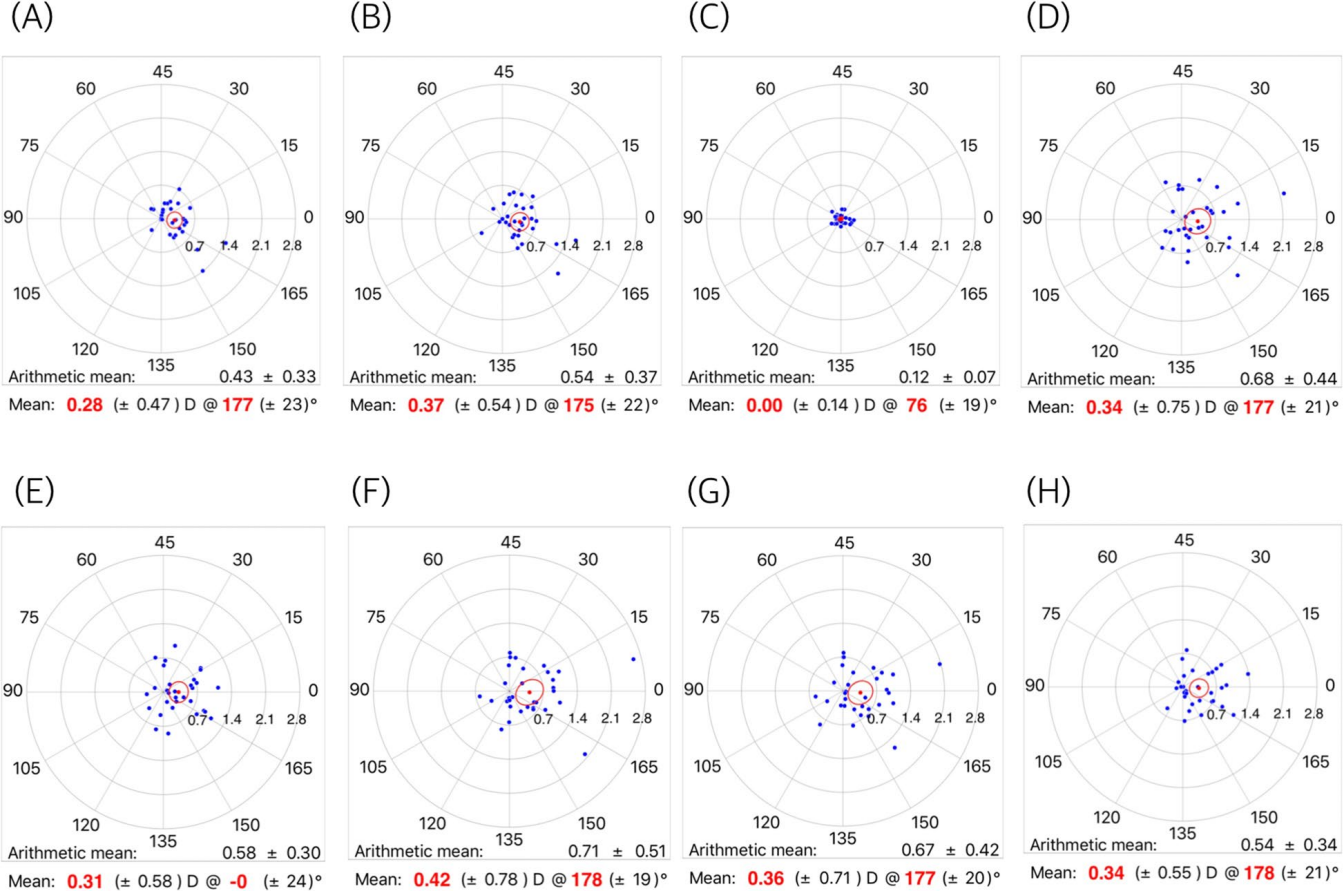
Mean surgically induced astigmatism (SIA) in superior incision (preoperative WTR astigmatism) group. **A** automated keratometer, **B** Scheimpflug anterior K, **C** Scheimpflug posterior K, **D** Scheimpflug EKR 3.0 mm, **E** Scheimpflug EKR 4.5 mm, **F** Scheimpflug TCRP 2.0 mm ring, **G** Scheimpflug TCRP 3.0 mm zone, and **H** Scheimpflug TCRP 4.0 mm zone

**Fig. 3:**
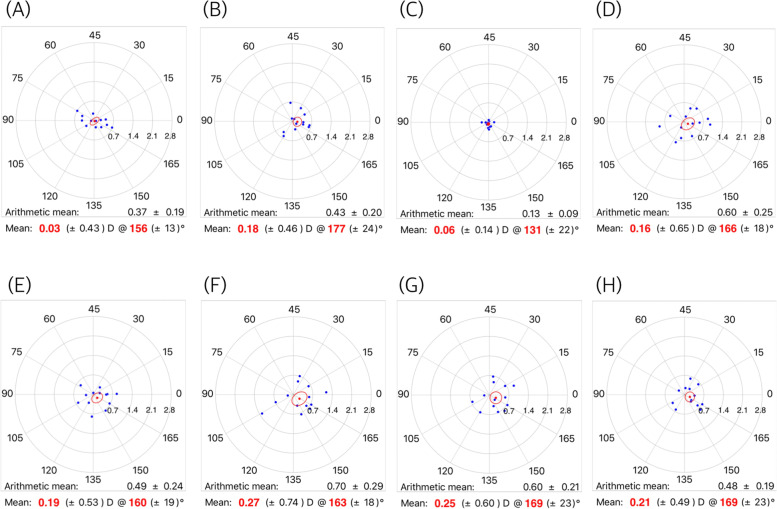
Mean surgically induced astigmatism (SIA) in superotemporal or superonasal incision (preoperative oblique astigmatism) group. **A** automated keratometer, **B** Scheimpflug anterior K, **C** Scheimpflug posterior K, **D** Scheimpflug EKR 3.0 mm, **E** Scheimpflug EKR 4.5 mm, **F** Scheimpflug TCRP 2.0 mm ring, **G** Scheimpflug TCRP 3.0 mm zone, and **H** Scheimpflug TCRP 4.0 mm zone

**Fig. 4:**
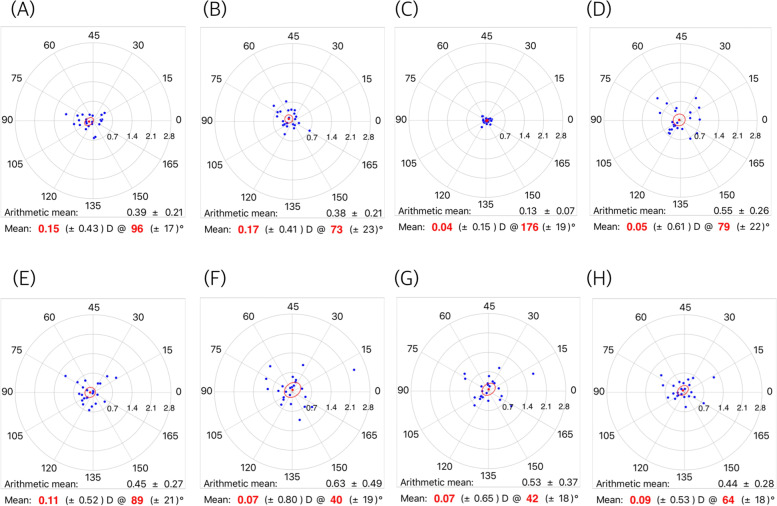
Mean surgically induced astigmatism (SIA) in temporal incision (ATR astigmatism) group. **A** automated keratometer, **B** Scheimpflug anterior K, **C** Scheimpflug posterior K, **D** Scheimpflug EKR 3.0 mm, **E** Scheimpflug EKR 4.5 mm, **F** Scheimpflug TCRP 2.0 mm ring, **G** Scheimpflug TCRP 3.0 mm zone, and (H) Scheimpflug TCRP 4.0 mm zone

Figure 1 shows the mean SIAs on the double-angle polar plots calculated by the aggregate analysis. All mean SIAs were less than 0.25 diopter. Figure 2 shows the mean SIAs in the preoperative WTR group. The mean SIAs determined by the automated keratometer and Scheimpflug anterior K were 0.28 diopter (axis: 177°) and 0.37 diopter (axis: 175°). The mean SIAs on the total cornea ranged from 0.31 to 0.42 diopter in WTR astigmatism. The mean SIAs in the oblique astigmatism group are shown in Fig. 3. The mean SIAs of the anterior corneal surface were 0.03 diopter (axis: 156°, for the automated keratometer) and 0.18 diopter (axis: 177°, for Scheimpflug anterior K). The mean SIAs of the total cornea ranged from 0.16 to 0.27 diopter. Figure 4 shows the results of the preoperative ATR astigmatism. In the ATR group, in particular, the mean SIA of the total cornea did not exceed the mean SIA of the anterior corneal surface (0.05 diopter, axis: 79 ° ~ 0.11 diopter, axis: 89°, for total cornea vs. 0.15 diopter, axis: 96 ° ~ 0.17 diopter, axis: 73°, for total cornea). The mean magnitude of SIA on the posterior corneal surface ranged from 0.12 to 0.13 diopter, but the mean SIAs on the double-angle polar plot did not exceed 0.1 diopter in the three subgroups (Figs. 2, 3 and 4).

## Discussion

This study demonstrated that ∆ J_0_ and ∆ J_45_ did not show any significant differences between the anterior cornea and the total cornea. Regardless of the preoperative steep meridian or location of the incision, the summated vector for the mean SIA on double-angle polar plots also did not show any significant differences.

The mean arithmetic SIA of the posterior cornea was 0.13 ± 0.08 diopter, and these results are similar to those in the study by Cheng et al. [26] However, our values were lower than those of the study by Nemeth et al. [12] The differences may be due to differences in inclusion criteria, as they included only patients with preoperative WTR astigmatism. The posterior cornea has a relatively steeper radius than the anterior cornea. Furthermore, when a more central location is created on the posterior cornea, a greater change is observed compared to a cut located more peripherally on the anterior cornea [26]. However, the difference in refractive indices between the cornea and aqueous humor is small [26]; hence, the posterior cornea induces minimal refractive astigmatism and can be ignored in the calculations of astigmatism [27]. In this study, the effect of the posterior corneal change on astigmatism was limited. Unlike the mean magnitude SIA on the posterior surface of the cornea, the summated vector mean SIA in double-angle polar plots did not exceed 0.1 diopters. The direction of the astigmatic change on the posterior surface was also the opposite direction to the astigmatic change on the anterior surface of the cornea (76° vs. 175–177°, for the WTR astigmatism group; 176° vs. 73–96°, for the ATR astigmatism group). Klijn et al. [28] found that the incision effect on the posterior corneal surface was of the same order of magnitude as the test-retest effect and concluded that the contribution of the posterior cornea to astigmatic change is limited. Kohnen et al. [29] also concluded that the SIA on the posterior corneal surface of a 2.2 mm femtosecond laser-assisted clear corneal incision was clinically insignificant. These results are contrary to the results of a recent study showing that posterior corneal astigmatism increased after the creation of a 1.8 mm steep meridian clear corneal incision [30]. Kim et al. [31] concluded that the direction of the astigmatism change in the posterior corneal surface after 2.2 mm temporal limbo-corneal incision was not uniform.

The mean magnitude of SIA on the total cornea was greater than the mean arithmetic SIA on the anterior corneal surface. Among the arithmetic mean SIAs on the total cornea, SIA on the more central cornea had a greater magnitude, and the TCRP 2.0 mm ring produced the greatest magnitude value of SIA. However, these results do not indicate that the amount of astigmatism reduction in the total cornea is greater than that in the anterior cornea. As the astigmatic changes in the total cornea occur in inconsistent directions, the mean change in the double-angle polar plot is not different from that of the anterior cornea. In Figs. 2, 3 and 4, the standard deviation of mean centroid SIA values on the total cornea ranged from 0.56 to 0.70 diopter (0.55–0.78 diopter in WTR astigmatism; 0.49–0.74 diopter in oblique astigmatism; 0.52–0.80 diopter in ATR astigmatism) and they were relatively greater than the standard deviation of the mean centroid SIA from the anterior corneal surface. (0.48–0.53 diopter in total 69 eyes; 0.47–0.54 diopter in WTR astigmatism; 0.43–0.46 diopter in oblique astigmatism; 0.41–0.43 diopter in ATR astigmatism).

A limitation of this study is that there is no clear indicator to determine the location of the steep meridian clear corneal incision. A rotating Scheimpflug camera provides various parameters for evaluating the astigmatism of the total cornea. The total corneal power can be calculated using a ray tracing method (TCRP) at 2.0, 3.0, 4.0, 5.0, 6.0, 7.0, and 8.0 mm and EKR at 2.0, 3.0, 4.0, 4.5, 5.0, and 6.0 mm. Savini et al. [32] concluded that a 3.0 mm zone TCRP and 2.0 mm ring TCRP accurately reflect surgically induced refractive changes in photorefractive keratectomy (PRK) and laser-assisted in situ keratomileusis (LASIK). They also found that 3.0 mm EKR and 2.0 mm TCRP induced the lowest median absolute errors when corneal measurements obtained by Pentacam HR were applied in cataract surgery [33]. Holladay et al. [21] demonstrated that EKR in the 4.5 mm zone yielded the highest correlation with subjective refraction compared to the historical method of K reading after LASIK and PRK. However, we performed a steep meridian incision, as determined by a Scheimpflug anterior K.

## Conclusion

In conclusion, our study demonstrated that, when considering the cornea as a whole, the SIA was not different from the SIAs with consideration of the anterior corneal surface only. A comparison between the steep meridian incision planned based on anterior corneal power measurements (automated keratometer or Scheimpflug anterior K) and the steep meridian incision based on total corneal power measurements (Scheimpflug EKR or TCRP) should be performed in future studies.

## Data Availability

The datasets used and/or analysed during the current study available from the corresponding author on reasonable request.

## Abbreviations

SIA: Surgically induced astigmatism
EKR: Equivalent keratometry reading
TCRP: Total corneal refractive power
WTR: With-the-rule
ATR: Against-the-rule
IOL: Intraocular lens
